# Model-Based Dose Selection of a Sphingosine-1-Phosphate Modulator, Etrasimod, in Patients with Various Degrees of Hepatic Impairment

**DOI:** 10.3390/pharmaceutics16121540

**Published:** 2024-12-01

**Authors:** Mohammed S. Alasmari, Faleh Alqahtani, Fawaz Alasmari, Abdullah Alsultan

**Affiliations:** 1 Drug and Poisoning Information Center, Security Forces Hospital, Riyadh 11481, Saudi Arabia; 2 Department of Pharmacology and Toxicology, College of Pharmacy, King Saud University, Riyadh 11451, Saudi Arabia; ffalasmari@ksu.edu.sa; 3 Department of Clinical Pharmacy, College of Pharmacy, King Saud University, Riyadh 11451, Saudi Arabia; absultan@ksu.edu.sa

**Keywords:** PBPK, etrasimod, liver impairment, precision dosing

## Abstract

Background/Objectives: Etrasimod is a newly FDA-approved Sphingosine-1-Phosphate modulator indicated for moderate and severe ulcerative colitis. It is extensively metabolized in the liver via the cytochrome P450 system and may accumulate markedly in patients with hepatic dysfunction, exposing them to toxicity. The aim of the current study is to utilize a physiologically-based pharmacokinetic modeling approach to evaluate the impact of hepatic impairment on the pharmacokinetic behavior of etrasimod and to appropriately select dosage regimens for patients with chronic liver disease; Methods: PK-Sim was used to develop the etrasimod PBPK model, which was verified using clinical data from healthy subjects and subsequently adapted to reflect the physiological changes associated with varying degrees of hepatic dysfunction; Results: Simulations indicated that hepatic clearance of etrasimod is clearly reduced in patients with Child–Pugh B and C liver impairment. Based on these findings, dosing adjustments were proposed to achieve therapeutic exposures equivalent to those in individuals with normal liver function. In the Child–Pugh B and C population groups, 75% and 62.5%, respectively, of the standard dose were enough to have comparable exposure to the healthy population. These adjusted dosages aim to mitigate the risk of drug toxicity while maintaining efficacy; Conclusions: The PBPK model provides a robust framework for individualizing drug therapy in patients with hepatic impairment, ensuring safer and more effective treatment outcomes. Further clinical studies are warranted to verify these dosing recommendations and to refine the model for broader clinical applications.

## 1. Introduction

Inflammatory bowel disease (IBD) has emerged, globally, as a major health concern, and its incidence is increasing steadily worldwide, particularly in newly industrialized nations [1,2]. Per 100,000 persons, annually, the incidence and prevalence were estimated to be 8 to 14 and 120 to 200, respectively, for UC, and 6 to 15 and 50 to 200, respectively, for CD [2]. Basically, IBD is an umbrella term for two chronic relapsing bowel inflammatory conditions: ulcerative colitis (UC) and Crohn’s disease (CD). In contrast to CD, which is characterized by a transmural inflammation that can potentially affect any segment of the whole gastrointestinal tract, spanning from the mouth to the perianal region, UC is characterized by inflammation that is confined to the mucosal and submucosal layers of the colon and rectum [3,4].

While the exact cause of IBD remains unknown, it has been proposed that there is a complex interplay between genetic, microbial, environmental, and immunological factors predisposing people to IBD [5,6]. The inheritance of susceptible genes and the presence of environmental triggers like microbial pathogens are principal factors in the pathogenesis of IBD. The dysregulation in the immunological response to pathogenic antigens leads to prolonged and uncontrolled activation and massive infiltration of immune cells, cytokines, and inflammatory mediators in the lining of the gastrointestinal tract, which is the main characteristic feature of IBD [7,8].

The ultimate therapeutic goal in the management of UC patients is to induce and maintain remission with the intent of histological healing [4]. For patients with mildly active UC, rectal enemas and oral 5-aminosalicylates are the recommended treatments for both induction and maintaining remission. In cases of moderate to severe active UC, recommended pharmacological options for inducing remission include oral systemic corticosteroids, infliximab (a TNF-blocking agent), adalimumab (a TNF inhibitor), tofacitinib (a Janus kinase inhibitor), and vedolizumab (a selective adhesion-molecule inhibitor) [4].

Despite the numerous therapeutic options for the treatment of UC, the remission rate was shown to gradually decrease over time in many patients [9]. Additionally, many treatments are biologics, which have the potential to increase the risk of infections, and generally, they are less preferable since they are available only in injectable dosage forms. Therefore, there is a need for other treatment options that are safe and effective and designed in more preferable dosage forms such as oral tablets.

Sphingosine 1 phosphate (S1P) is an inflammatory mediator produced as a biological end product from the turnover of cell membrane-derived sphingolipids into sphingosine in the presence of ceramidase as a catalytic enzyme, and sphingosine is then phosphorylated into S1P by a kinase enzyme [10]. The biological effect of S1P is exerted via binding to a specific receptor (S1PR) that is ubiquitously expressed on the surface of different inflammatory cells including macrophages, neutrophils, eosinophils, dendritic cells, monocytes, and natural killer cells. S1PR belongs to the class of G-protein-coupled receptors (GPCRs) and has five subtypes (S1PR_1–5_). The activation of this receptor by S1P is critically involved in the pathological mechanism of multiple immune-mediated conditions, including UC [10].

Etrasimod is an oral selective S1P receptor modulator approved recently (12 October 2023) by the Food and Drug Administration (FDA) to treat moderately to severely active ulcerative colitis in adults [11]. Etrasimod induces remission in UC patients by inhibiting the differentiation, trafficking, and migration of inflammatory cells into peripheral tissues, including the gastrointestinal tract. The efficacy and tolerability of etrasimod for treating moderate to severe UC have been demonstrated in phase 2 and 3 clinical trials [12,13,14]. A significant improvement has been achieved in the outcomes of interest including histological appearance and symptomatic remission. Etrasimod was developed as a small molecule with low molecular weight in a tablet dosage form to be suitable for oral administration routes [15].

The absorption of etrasimod is slightly rapid with a median time to peak of 4 h (range of 2 to 8 h) [11]. Etrasimod is highly distributed in peripheral body tissues and highly protein-bound with values of 66 L and 97.9%, respectively. Dose proportionality was observed in the dosage range of 0.7 mg to 2 mg with a mean half-life of about 30 h, and the steady state was achieved at day 7. Etrasimod is extensively metabolized in the liver with the involvement of multiple cytochrome P450 enzymes and subsequently excreted into feces through the biliary system, with negligible contribution to renal clearance. In vitro reactions indicated that cytochrome P450 2C8 (CYP2C8), 2C9 (CYP2C9), and 3A4 (CYP3A4) are the predominant enzymes involved in the metabolism of etrasimod, while cytochrome P450 2C19 (CYP2C19) and 2J2 (CYP2J2) contributed minimally [16]. The fraction metabolized (fm), indicating the proportion of a drug’s metabolism attributed to specific enzymes, is as follows for these enzymes in etrasimod metabolism: CYP2C8 (0.38), CYP2C9 (0.37), CYP3A4 (0.22), CYP2C19 (0.01), and CYP2J2 (0.01) [16]. A high fm value indicates that a significant portion of the drug’s metabolism depends on a single enzyme, making the drug more susceptible to changes in clearance if that enzyme is inhibited (e.g., by a co-administered drug) or impaired (e.g., in hepatic disease). This can lead to increased drug exposure and a higher risk of adverse effects. According to the FDA guidance on drug interaction studies, an fm value exceeding 0.25 is considered significant, as it indicates that a single enzyme contributes substantially to a drug’s metabolism, increasing the likelihood of clinically relevant drug-drug interactions or changes in clearance [17].

Liver functionality is a very important determinant of the kinetic behavior of drugs, and several pharmacokinetic parameters are significantly impacted in patients with liver dysfunction [18]. Therefore, drugs that are mainly detoxified in the liver such as etrasimod should be closely monitored in such patient populations, and doses should be selected appropriately according to the health status of the liver to avoid toxicity. Since that etrasimod is eliminated mainly via hepatic metabolism, its exposure therefore has the potential to be altered in patients with liver impairment.

As a strategy of model-informed drug development (MIDD), physiologically-based pharmacokinetic (PBPK) modeling has enhanced the discovery, development, and assessment of many drugs and biologics [19,20]. The PBPK modeling is a quantitative mechanistic approach through which biological processes, including absorption, distribution, metabolism, and elimination (ADME), are described mathematically to predict the pharmacokinetic parameters of drugs. This mathematical modeling tool is being widely used to evaluate the effect of several intrinsic and extrinsic factors (e.g., renal and hepatic impairment, drug interactions, pregnancy) on drug exposure among different patient populations. In hepatic and renal impairment populations, it has been shown that PBPK modeling adequately predicts drug exposure based on the severity of the diseases [21]. PBPK models can be built by the integration of drug-dependent parameters (e.g., lipophilicity, molecular weight, pKa, solubility) and biological system-dependent parameters (e.g., organ volume, enzyme abundance) in a specific PBPK platform [22].

While the etrasimod-approved label specifies that no dose adjustment is needed in patients with mild and moderate hepatic impairment, PBPK modeling provides a mechanistic approach to explore the pharmacokinetics in this population in a more nuanced manner [21]. It considers the physiological changes in hepatic function and drug metabolism that might not be captured by traditional approaches. PBPK models can simulate drug behavior under various physiological conditions, enabling personalized insights, especially in populations where data might be limited or not fully represented in clinical trials.

Clinical studies in hepatic impairment are often conducted with limited sample sizes and might not capture the full variability within this population. The PBPK model complements these data by providing a tool for simulating a wide range of scenarios, including the impact of severe hepatic impairment [21]. Although it is not recommended for etrasimod to be taken in patients with severe hepatic impairment, PBPK models can be useful in exploring borderline cases and can help reinforce dosing decisions based on mechanistic insights.

In this study, therefore, we utilized the PBPK modeling and simulation method to evaluate the effect of hepatic impairment on the pharmacokinetic behavior of etrasimod to appropriately select dosage regimens for patients with hepatic impairment and to inform the study design for future clinical trials.

## 2. Materials and Methods

### 2.1. Software and Information Resources

For developing the etrasimod PBPK model, we utilized the PK-Sim software (Version 11.2; Bayer Technology Services, Bayer AG, Leverkusen, Germany), which is freely provided by Bayer AG through the Open System Pharmacology (OSP) Suite (https://www.open-systems-pharmacology.org (accessed on 24 November 2024)). The PK-Sim is a population-based simulator consisting of several building blocks through which physicochemical properties of the drug can be integrated with anatomical and physiological properties of the biological system to simulate concentration–time profiles and predict the pharmacokinetic parameters of drugs [22]. Structurally, the PBPK model is represented by interconnected physiological compartments representing different body organs and they are connected with each other by the circulatory system. The software integrates detailed physiological information with drug-related parameters to simulate the kinetic behaviors of drugs in humans. In addition, it allows for modeling interactions between drugs by considering the impact of a perpetrator on the pharmacokinetics of a victim drug.

The WebPlotDigitizer^®^ software version 4.6 was used to extract the observed data from concentration–time profiles of clinical pharmacokinetic trials [23]. Human Metabolome [24], Drug Bank [25], and Lexicomp (https://online.lexi.com/lco/action/home. Accessed on 15 February 2024) databases were used to retrieve the required drug-specific input data. Predicted pharmacokinetic parameters were compared with the values reported in the clinical trials (Table 1), which were originally estimated using noncompartmental analysis in the corresponding trials. We compared plasma concentrations, the area under the curve from time 0 to infinity (AUC__inf_), and the maximum concertation (C_max_). The R programming language version 4.3.3 and RStudio version 2024.04.1+748 were used for plotting the concentration versus time profiles and to visually represent the observed to predicted ratio.

### 2.2. Model Development

**Figure 1:**
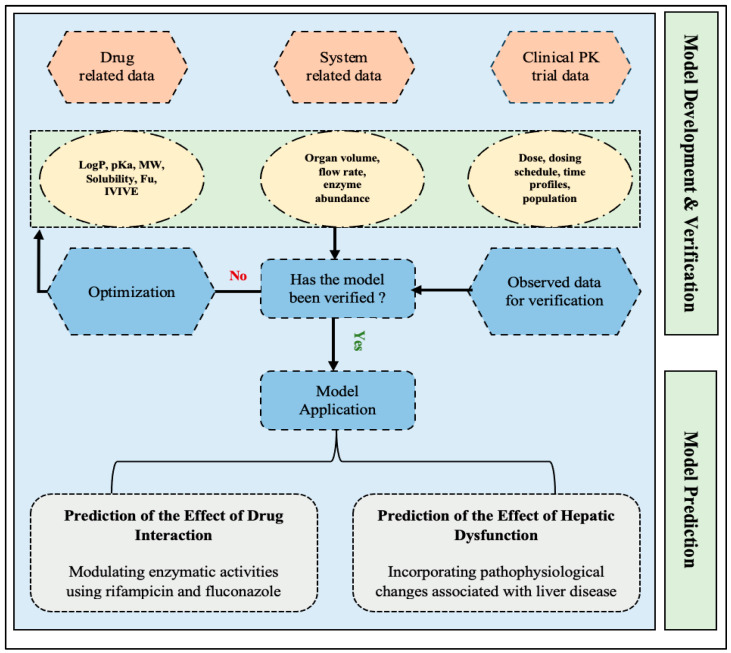
General workflow of the etrasimod PBPK model development and verification.

Initially, we conducted an extensive literature search through multiple databases to gather the required information for model development and verification. The general workflow of model building and verification strategy was represented in Figure 1, and input data that were used to inform the PBPK model parameters were summarized in Table 2.

Briefly, physicochemical properties of etrasimod including molecular weight, lipophilicity, ionization, fraction unbound, and solubility were collected and incorporated into the software to inform model input parameters. Intestinal permeability was directly calculated by PK-Sim based on molecular weight and lipophilicity for modeling the absorption phase [30,31]. A Weibull function was assumed to quantify dissolution behavior with subsequent optimization of dissolution time to be consistent with reported information. The default value for dissolution time (the time required for 50% of the administered dose to be dissolved) is 240 min and the optimized value is 120 min, which is in line with the fact that etrasimod is absorbed rapidly with a median time to peak of 2 to 8 h [15]. For distribution modeling, the Rodger and Roland method was used for the calculation of the partition coefficient and PK-Sim standard method was used for the calculation of cellular permeability.

To estimate hepatic clearance of etrasimod in our PBPK model, we used in vitro–in vivo extrapolation (IVIVE) data published by Lee et al. (2023) [16]. This study measured intrinsic clearance values using both human liver microsome (HLM) and recombinant CYP enzymes (rCYP) for CY2C8, CYP2C9, CYP3A4, CYP2C19, and CYP2J2, the primary enzymes involved in etrasimod metabolism. The original data of intrinsic clearance were provided in units of µL/min/mg, requiring conversion to µL/min/pmol for compatibility with PK-Sim’s scaling framework. The units were converted by using the enzyme abundance in pmol/mg that was provided in the study (Table 2) [16]. The following equation was used for unit conversion, as follows:

Cl_int_ in µL/min/pmol = (Cl_int_ in µL/min/mg)/(rCYP abundances in pmol/mg).

**Table 2:** Input data used to inform model parameters.

Parameter	Unit	Input Value	Reported Value	Reference
**Physicochemical properties**				
Molecular weight	g/mol	457.493	457.493	[24]
Effective molecular weight	g/mol	406.5		PK-Sim
Lipophilicity	Log	5.73	5.73, 6.45	[24]
pKa		4.26	4.26	[24]
Solubility	mg/mL	0.000477	0.000477	[25]
Plasma protein binding	%	97.9	97.9	[25]
**Absorption**				
Intestinal permeability	cm/min	0.02		Calculated
Dissolution model	Weibull			PK-Sim
Dissolution time (50% dissolved)	min	120		Optimized
**Distribution**				
Partition coefficient model	Rodger and Roland			PK-Sim
Cellular permeability model	PK-Sim			PK-Sim
Fraction unbound	%	2.10		[25]
Blood to plasma ratio		0.66	0.66	[16]
**Enzymatic biotransformation** ***Process type:*** In vitro metabolic rate in presence of recombinant enzymes				
CYP2C8	µL/min/pmol	0.01157		[16]
CYP2C9	µL/min/pmol	0.08525		[16]
CYP3A4	µL/min/pmol	0.01640		[16]
CYP2J2	µL/min/pmol	0.01872		[16]
CYP2C19	µL/min/pmol	0.01331		[16]
**Parameterization of enzyme inhibition process**				
Ki for fluconazole-CYP3A4 complex	µmol/L	10.7		[32]
Ki for fluconazole-CYP2C9 complex	µmol/L	19.60		[33]
Ki for fluconazole-CYP2C19 complex	µmol/L	1.74		[33]
**Parameterization of enzyme induction process**				
EC50 for rifampicin-CYP3A4 complex	µmol/L	0.34	0.34	[34]
Emax for rifampicin-CYP3A4 complex		9	9	[34]
EC50 for rifampicin-CYP2C8 complex	µmol/L	0.34	0.34	[35]
Emax for rifampicin-CYP2C8 complex		3.2	3.2	[35]

### 2.3. Etrasimod PBPK Model Development and Verification in Adult Healthy Population

A dataset for the adult healthy population based on physiological characteristics has been provided by the PK-Sim and was utilized for modeling etrasimod pharmacokinetics in a typical average adult population. The PK-Sim includes default values for physiological parameters such as organ volumes, blood flows, tissue, and blood compositions. With average weight and age of 73 kg and 30 years, respectively, a virtual adult healthy population of 100 virtual individuals was created. For all simulations, time profile analysis was conducted, and distribution statistics of mean and 5th to 95th prediction range were selected to display.

The predictive performance and adequacy of the model were verified initially based on visual inspection of concentration–time profiles with respect to the deviation of observed data points from the simulated mean. A goodness of fit plot was used to assess how well simulated and observed concentrations are fit. In addition, we compared predicted and observed values of AUC__inf_ and C_max_ using the average fold error (AFE) methods, by calculating the ratio of predicted to observed values. The standard two-fold error range was acceptable to verify the model’s predictability. This range has been widely applied and accepted in the field of drug discovery and development as mentioned previously [36,37].

### 2.4. Modeling the Effect of Drug Interactions on the Etrasimod Pharmacokinetic

Etrasimod is a substrate for multiple CYP enzymes and, therefore, its metabolism can be affected if it is being taken concurrently with drugs that modulate the activity of these enzymes. Fluconazole is an antifungal agent used commonly to treat various fungal infections. Due to its inhibitory effects on multiple cytochrome P450 enzymes including CYP2C9, CYP 2C19, and CYP3A4 [32,33], fluconazole has the potential for interaction with etrasimod. Rifampicin is a potent inducer for CYP3A4 and CYP2C8 and may affect the exposure of etrasimod [34,35].

Therefore, the final verified model of etrasimod was used to assess the effect of drug interaction with these drugs. Hepatic metabolism of etrasimod was inducted and inhibited using rifampicin and fluconazole models, respectively, which were developed previously and have been made freely available through the repository of the OSP [38] (https://github.com/Open-Systems-Pharmacology. Accessed on 1 March 2024).

These models were verified to be used as perpetrators for evaluating the effect of drug interactions on biological exposure of CYP enzyme substrates [39]. The inhibitory effects of fluconazole on CYP3A4, CYP2C9, and CYP2C19 were parameterized in terms of dissociation constants (Ki) for the complex of fluconazole with these enzymes, as described previously (Table 2) [32,33]. The induction of CYP3A4 and CYP2C8 by rifampicin was parameterized in terms of EC50 and Emax, as described previously [34,35]. Emax for the induction process represents the maximum in vivo induction effect, while EC50 represents the concentration of the inducer at which 50% of the maximum induction efficacy is achieved. The predictive performance of the model was verified based on the results from the phase one study [27].

### 2.5. Modeling the Effect of Hepatic Impairment on the Etrasimod Pharmacokinetic

In order to predict etrasimod exposure in the hepatically impaired patient population, we have adjusted the model for the pathophysiological changes associated with each class of the Child–Pugh grading system of chronic liver disease [40]. According to this grading system, patients were categorized into three categories based on the severity of the liver disease: grade A (<7 points), grade B (7–9 points), and grade C (10–15 points). The criteria for the Child–Pugh grading classification included total bilirubin, albumin, ascites, encephalopathy, and prothrombin time. We have obtained and integrated data on changes in liver volume, hepatic blood flow, albumin, alpha 1 acid glycoprotein, hematocrit, glomerular filtration rate (GFR), and hepatic enzymes activities associated with each Child–Pugh class (Table 3) as previously described [41,42].

The ability of the model to predict the impact of liver impairment on the exposure was qualified based on the information from FDA approved label of etrasimod [11]. The FDA-approved label reported an increase in the total AUC__inf_ by 13%, 29%, and 57% (with respect to control healthy values) in patients with mild, moderate, and severe hepatic impairment, respectively (LABEL (fda.gov)) [11]. This was based on a pharmacokinetic study of etrasimod in patients with mild, moderate, and severe hepatic impairment [43]. However, because concentration–time profiles for hepatic impairment were not available, we ran the simulation without observed concentration data and then compared the predicted exposure with the reported value.

**Table 3:** Pathophysiological alterations associated with chronic liver disease.

Model Parameter	Severity of Liver Disease		
	Child–Pugh A	Child–Pugh B	Child–Pugh C
Portal vein blood flow ^a^	0.40	0.36	0.04
Hepatic arterial blood flow ^a^	1.3	2.3	3.4
Renal blood flow ^a^	0.88	0.65	0.48
Cardiac index ^a^	1.11	1.27	1.36
Blood flow in other organs ^a^	1.75	2.25	2.75
Albumin ^a^	0.81	0.68	0.50
Alpha-1-acid glycoprotein ^a^	0.60	0.56	0.30
Hematocrit ^a^	0.39	0.37	0.35
Functional liver mass ^a^	0.69	0.55	0.28
GFR ^a^	1	0.70	0.36
CYP3A4 activity ^b^	1	0.40	0.40
CYP2J2 activity ^b^	1	1	1
CYP2C8 activity ^c^	0.69	0.52	0.33
CYP2C9 activity ^c^	0.69	0.52	0.33
CYP2C19 activity ^c^	0.32	0.26	0.12

^a^ Fraction of control values as described by Edginton et al. (2008) [42]. ^b^ Values as described by Willmann et al. (2021) [44]. ^c^ Fraction of control values as described by Johnson et al. (2010) [41].

### 2.6. Dosing Adjustment of Etrasimod Based on the Severity of the Liver Impairment

We tailored dosing regimens for etrasimod to individual patients’ specific Child–Pugh scores, ensuring that drug exposure in each population is within the desired therapeutic range. We have selected the dosing regimens that were expected to achieve the target exposure levels comparable to healthy individuals. The extent of drug exposure is often quantified by the AUC__inf_, since maintaining a specific drug level is crucial for efficacy and safety [45]. Thus, Box and Whisker plots were used to visually represent the variability in the AUC__inf_ of etrasimod across various levels of hepatic impairment. For each group of the hepatically impaired population, we created a Whisker box plot to visualize the distribution of AUC__inf_, as an indicative of etrasimod exposure, and we gradually reduced the doses accordingly, to have comparable drug exposure to healthy individuals.

## 3. Results

### 3.1. Development of the Etrasimod PBPK Model in an Adult Healthy Population

**Figure 2:**
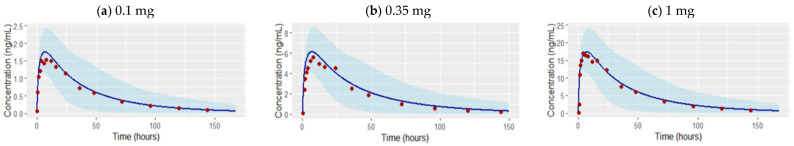

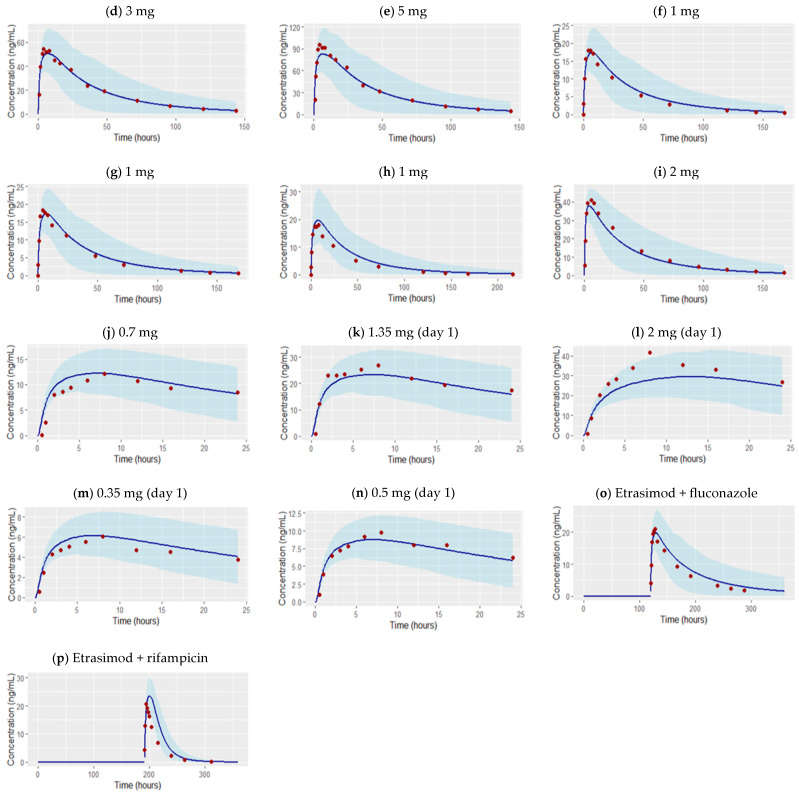
Observed (symbols) and PBPK simulated (lines) plasma concentration–time profiles of etrasimod in adult healthy population after single-dose regimens. The sky-blue shaded area represents the 5th–95th percentile prediction interval from the PBPK model. (**a**) In total, 0.1 mg single oral dose [28], (**b**) 0.35 mg single oral dose [28], (**c**) 1 mg single oral dose [28], (**d**) 3 mg single oral dose [28], (**e**) 5 mg single oral dose [28], (**f**) 1 mg single oral dose cohort A [27], (**g**) 1 mg single oral dose cohort B [27], (**h**) 1 mg single oral dose [26], (**i**) 2 mg single oral dose [16], (**j**) day 1 of multiple fixed 0.7 mg oral dose [28], (**k**) day 1 of multiple fixed 1.35 mg oral dose [28], (**l**) day 1 of multiple fixed 2 mg oral dose [28], (**m**) day 1 of multiple escalated 0.35 mg oral dose [28], (**n**) day 1 of multiple escalated 0.5 mg oral dose [28], (**o**) simulated time profiles of etrasimod taken concurrently with fluconazole [27], (**p**) simulated time profiles of etrasimod taken concurrently with rifampicin [27].

**Figure 3:**
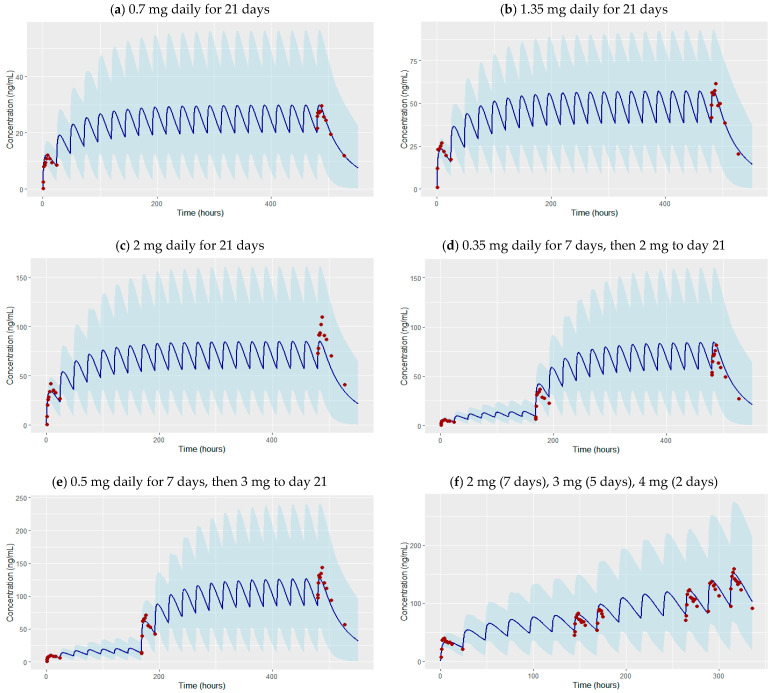
Observed (symbols) and PBPK simulated (lines) plasma concentration–time profiles of etrasimod in adult healthy population after multiple fixed and escalated oral dosing regimens. The sky-blue shaded area represents the 5th–95th percentile prediction interval from the PBPK model. (**a**) In total, 0.7 mg daily for 21 days [28], (**b**) 1.35 mg daily for 21 days [28], (**c**) 2 mg daily for 21 days [28], (**d**) 0.35 mg daily for 7 days, then 2 mg to day 21 [28], (**e**) 0.5 mg daily for 7 days, then 3 mg to day 21 [28], (**f**) 2 mg daily for 7 days, then 3 mg from day 8 to 12, then 4 mg from day 13 to 14 [29].

The developed PBPK model that incorporates drug-specific ADME properties and physiological system parameters reliably predicted the time course of etrasimod concentrations and captured the graphically observed concentrations from different clinical trials. The concentration versus time profiles presented in Figure 2 and Figure 3 illustrate the performance of the developed PBPK model in predicting etrasimod concentrations over time for several doses, including both single and multiple dosing regimens. Most of the observed data fall within the model’s 5th to 95th prediction interval.

**Figure 4:**
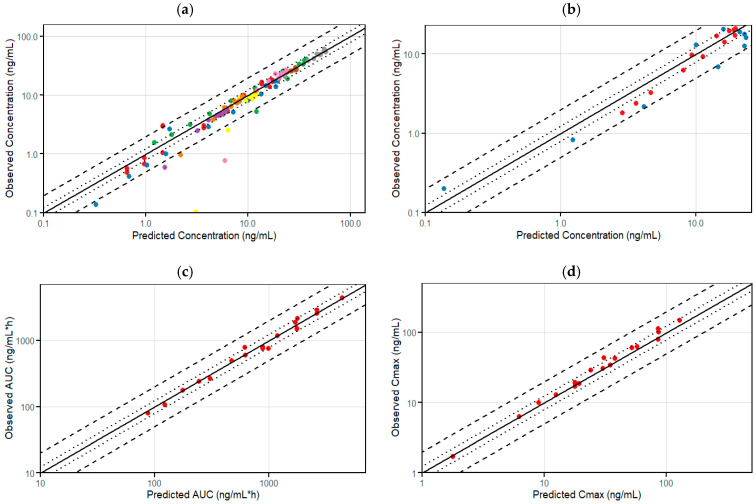
Goodness-of-fit plots for model verification. (**a**) Observed versus predicted concentrations for all time points after single and multiple dosing regimens. Each data point represents an individual concentration measurement at a specific time, highlighting the agreement between observed and predicted values across the concentration–time profile. (**b**) Observed versus predicted concentrations for all time points after drug interactions (blue circles with rifampicin, and red circles with fluconazole). Each data point represents an individual concentration measurement at a specific time, highlighting the agreement between observed and predicted values across the concentration–time profile. (**c**) Observed versus predicted AUC values. (**d**) Observed versus predicted C_max_ values. Each panel includes a line of unity (solid diagonal line), indicating perfect agreement between observed and predicted values. Two sets of dotted and dashed lines are included to represent 1.25-fold and 2-fold error limits, respectively, providing benchmarks for acceptable prediction accuracy.

Moreover, a goodness-of-fit plot comparing observed versus predicted concentrations for several doses of etrasimod after single and multiple dosing regimens confirms the accuracy and reliability of the PBPK model (Figure 4a). Most of the data points fall within the 2-fold limit, suggesting that the PBPK model’s predictions are within an acceptable error range, reinforcing its validity and precision.

**Figure 5:**
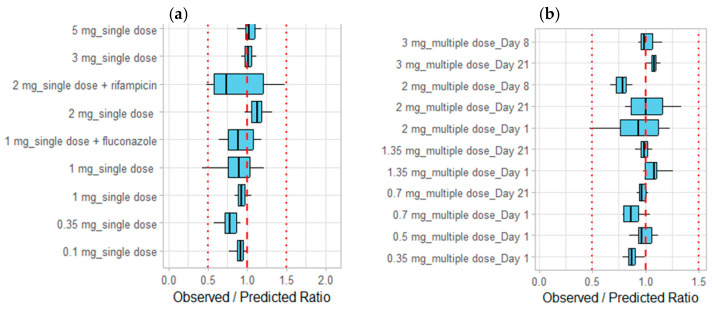

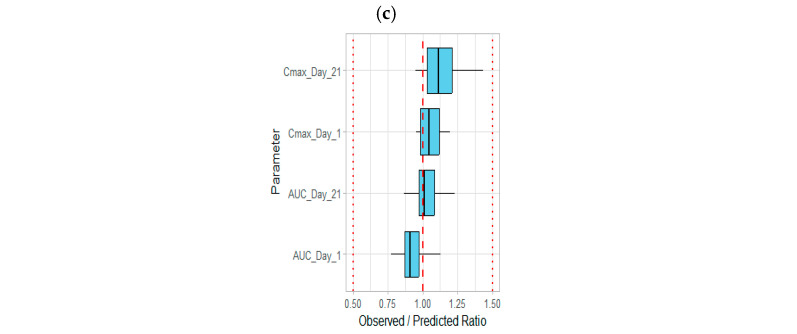
Observed-to-predicted ratios are presented for etrasiomd concentrations after single dosing regimens (**a**), multiple dosing regimens (**b**), and for PK parameters AUC__inf_ and C__max_ (**c**). Box and Whisker plots were used to depict the distribution of the ratios. The dashed line at an observed/predicted ratio of 1, indicating perfect agreement between observed and predicted values; and two dotted lines at 0.5 and 1.5, representing the bounds for a 0.5 to 1.5 fold error range. The distribution of the Box and Whisker plots relative to these lines provides a visual assessment of the model’s predictive performance, with data points falling within the 0.5- to 1.5-fold range indicating acceptable predictive accuracy.

The goodness-of-fit plots for the PK parameters (AUC__inf_ and C_max_) further verify the model, showing that the predicted PK parameters closely match the observed values, thereby confirming the robustness of the PBPK model (Figure 4c,d). The overall alignment and distribution of data points across plots offer a comprehensive evaluation of the model’s goodness-of-fit and predictive capabilities. Additionally, the ratios of the observed to predicted values for concentrations and PK parameters of etrasimod were depicted visually for further model qualification (Figure 5).

### 3.2. Modeling the Effect of Drug Interaction on the Etrasimod Exposure

Based on the results from a clinical pharmacokinetic study comparing etrasimod concentrations in the presence and absence of liver enzymes inhibitor and inducers [27], we assessed the performance of the developed PBPK model in describing concentration–time profiles of etrasimod taken concurrently with rifampicin and fluconazole as hepatic enzymes inducer and inhibitor, respectively.

The effect of drug interactions on the concentration–time profile of etrasimod has been predicted precisely by the developed etrasimod PBPK model (Figure 2o,p). The simulated concentration–time profiles for etrasimod as a victim for drug interaction were in a good agreement with the clinically observed profiles, given that observed data fall within the model’s 5th to 95th prediction interval. This suggests a high degree of accuracy and reliability in the model’s predictive capability. The goodness of fit plot comparing observed versus predicted concentrations of etrasimod taken concurrently with fluconazole and rifampicin illustrate the verification of the developed model to predict the effect of drug interactions on the etrasimod exposure (Figure 4b).

The ratio of observed versus predicted values of etrasimod concentrations in the presence of hepatic enzymes inducer and inhibition was within the accepted error range of 0.5- to 1.5- fold (Figure 5a).

**Figure 6:**
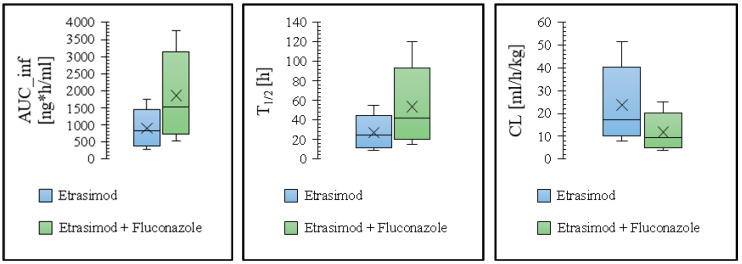

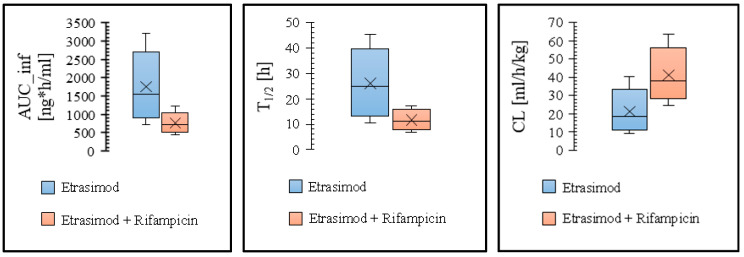
Box and Whisker plots depicting the predicted PK parameters for etrasimod with and without the perpetrators. The lower Whisker represents the minimum value, the lower edge of the box indicates the 25th percentile, the line within the box marks the median, the upper edge of the box shows the 75th percentile, the upper Whisker represents the maximum value, and the × sign represent the mean.

The changes in different pharmacokinetic parameters for etrasimod as a victim of drug interaction were depicted in Figure 6, and the predicted and observed ratios (in the presence/absence of fluconazole and rifampicin) of the AUC__inf_, C_max_, and T½ are represented in Table 4. The following equations were used to calculate drug interaction ratios for the PK parameters [46].

DDI AUC ratio = (AUC victim drug during co-administration)/(AUC victim drug)

DDI C_max_ ratio = (C_max_ victim drug during co-administration)/(C_max_ victim drug)

DDI T½ ratio = (T½ victim drug during co-administration)/(T½ victim drug)

### 3.3. Modeling the Effect of Hepatic Impairment on the Etrasimod Pharmacokinetics

**Figure 7:**
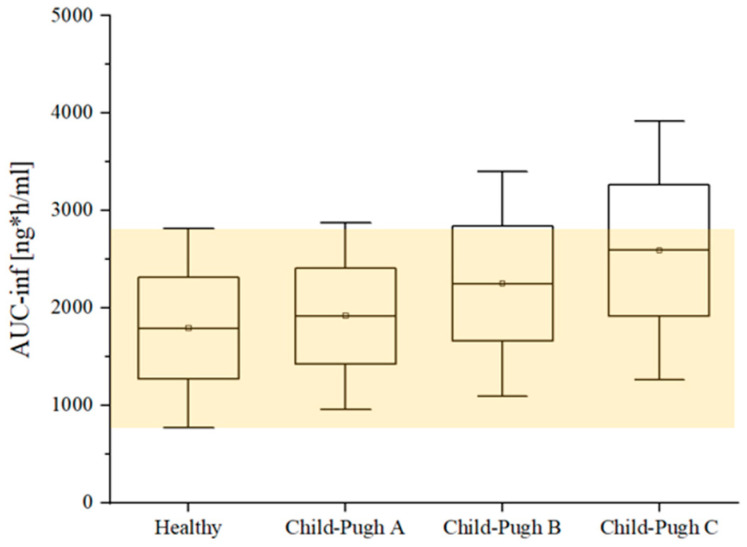
Box and Whisker plot illustrating the predicted drug exposure stratified by liver impairment severity (Child–Pugh A, B, and C). The boxes represent the mean values, and the Whiskers indicate the standard error (SE). This analysis demonstrates the impact of different levels of hepatic impairment on etrasimod exposure.

As indicated in Table 5, the current PBPK model possesses sufficient capability to predict etrasimod exposure in patients with liver impairment, according to the severity of the disease. The predicted to observed values of AUC__inf_ for Child Pugh groups A, B, and C were 1.11, 1.13, and 1.07, respectively. These values indicate good predictability for the developed PBPK model. In proportion to healthy individuals, the effect of hepatic dysfunction on etrasimod exposure is illustrated in Figure 7.

**Figure 8:**
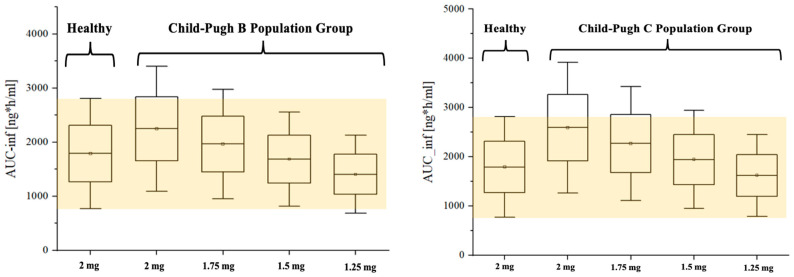
Dosing optimization for etrasimod in Child–Pugh B (**left panel**) and Child–Pugh C (**right panel**) population groups. The figure represents the simulated exposures after gradually reducing the standard dose (2 mg) of etrasimod. We found that 1.5 mg and 1.25 mg in Child–Pugh B and Child–Pugh C population groups, respectively, provide comparable exposure to healthy populations.

For dosing optimization, Box and Whisker plot analysis revealed that 1.5 mg (75% of the dose given to the healthy population) in Child–Pugh-B group (Figure 8, left panel) and 1.25 mg (62.5% of the dose given to healthy population) in Child–Pugh C group (Figure 8, right panel) provided the closet exposure to the 2 mg single dose in healthy population, where both means and SEs were included in the range of healthy population.

## 4. Discussion

In spite of the availability of numerous approved medications for UC, a significant number of patients do not respond to the prescribed drugs as expected clinically [13,14]. In addition to their association with developing serious infections, most of the currently available treatments for UC are administered parenterally; the route that is not preferred by patients. Therefore, etrasimod was developed to overcome the drawbacks of the previous generation of UC medications. However, due to insufficient clinical data supporting the appropriate dosing regimen in patients with chronic liver disease, it was necessary to employ the PBPK modeling approach to predict its kinetic behavior in this patient population.

The advantage of the PBPK modeling and simulation allows for extrapolation of pharmacokinetic data from healthy to non-healthy individuals (e.g., chronic liver disease), taking into account the anatomical and physiological considerations of the human body [21,47,48]. Since etrasimod is a hepatically cleared medication and its exposure may be altered with impairment in liver functions, in this study, we used the PBPK modeling and simulation technique to predict its pharmacokinetic profile in patients with liver impairment to support the dose selection process. We developed this PBPK model for etrasimod based on recommendations that advocate a stepwise design, starting from establishing the model on a healthy population, and subsequently incorporating the physiological values differences arising from liver disease [49].

Etrasimod is pharmaceutically supplied as a tablet dosage form for oral administration. Therefore, we started by simulating the absorption phase directly through calculation of intestinal permeability with taking into account physicochemical properties of the drug [31]. In addition, physicochemical properties were used to calculate partition coefficients for modeling the distribution phase of etrasimod. For metabolic biotransformation, the model was parameterized according to the in vitro metabolic rate in the presence of recombinant enzymes [16]. The ability of the developed model to predict the concentration–time course of etrasimod in the body was verified given that observed concentrations were contained within the predicted range. In addition, the model performance was verified given that the ratios of the observed to predicted values of pharmacokinetic parameters were within acceptable range of the fold error. These criteria indicate that the developed model for healthy population is verified to be used for scaling into other patient population such as those with hepatic dysfunction.

Extrapolation of the model to the hepatically impaired patient population was verified based on what has been reported in the FDA label of etrasimod [11]. It has been mentioned that there are 13%, 29%, and 57% increases in the AUC of etrasimod in patients with mild, moderate, and severe hepatic impairment, respectively. Our model was able to precisely predict these gradual increases in the exposure corresponding with deterioration in the liver metabolic functions. Generally, the performance of this model supports the applicability of the PBPK modeling and simulation approach in the dose selection of etrasimod in patients with liver diseases during clinical development.

While specific toxic and therapeutic levels are not provided, the research suggests that 2 mg once daily of etrasimod is both efficacious and well-tolerated in healthy normal populations in clinical settings [12]. According to the FDA Drug Label [11], oral administration of etrasimod to male rats at doses of 25, 100, or 200 mg/kg/day from the pre-mating period through mating showed no negative impact on fertility, with exposure levels reaching up to 467 times the maximum recommended human dose (MRHD) of 2 mg, based on AUC comparisons. Similarly, in female rats, doses of 1, 2, or 4 mg/kg/day given from pre-mating to implantation had no detrimental effects on fertility, with exposures up to 21 times the MRHD. In addition, preclinical long-term carcinogenicity studies showed that mice treated with etrasimod at doses of 2, 6, or 20 mg/kg/day for up to 104 weeks exhibited an increased incidence of hemangiosarcoma and hemangioma in both sexes at doses of 6 and 20 mg/kg/day, corresponding to exposures approximately 42 and 121 times the MRHD of 2 mg, based on AUC comparisons [11].

Studies have indicated that liver dysfunction is one of the most important factors to consider during dosage adjustment to avoid drug accumulation in the body and subsequent toxicity [18,50,51]. The bioavailability of etrasimod and its elimination largely depend on the liver’s functional efficiency. Etrasimod undergoes extensive oxidative metabolism in the liver via cytochrome P450 system, primarily CYP3A4, CYP2C8, and CYP2C9 [16]. Intuitively, the inefficiency of these enzymes for any reason can significantly impact etrasimod pharmacokinetics. Therefore, it is worth for dosing regimens to be adjusted appropriately especially in patients with severe hepatic impairment, signifying the importance of the PBPK modeling and simulation in the individualized medicine.

As per the FDA label, in patients with mild to moderate hepatic impairment (Child–Pugh A and B), etrasimod exposure was comparable to those with normal hepatic function [11]. However, its exposure was elevated in individuals with severe hepatic impairment (Child–Pugh C) compared to those with normal hepatic function. Therefore, the FDA label recommends against the use of etrasimod in patients with severe hepatic impairment (as this may lead to significantly increased drug exposure) and states that no dosage adjustment is necessary for those with mild to moderate hepatic impairment. These recommendations reflect the importance of hepatic function in etrasimod metabolism and the need for caution in populations with severe impairment to avoid potential toxicity or adverse effects. The ultimate goal of this work is to propose an appropriate dosing regimen for patients with severe hepatic impairment using the PBPK approach as it provides a mechanistic framework to predict drug exposure in this population, enabling informed dosing recommendations that address the limitations of current knowledge. Although no dose adjustment is required for patients with mild to moderate hepatic impairment, PBPK modeling offers a mechanistic method to investigate the pharmacokinetics in this population more comprehensively. This approach accounts for physiological alterations in hepatic function and drug metabolism that might not be captured by traditional approaches.

One of the most common applications of the PBPK modeling is to estimate the impact of drug interaction on the bioavailability of drugs [20]. Considering that etrasimod is a substrate for several hepatic CYP450 enzymes, it was also necessary to test the ability of the PBPK model to predict the impact of induction and inhibition of these enzymes by perpetrators on the pharmacokinetic of etrasimod as a victim. Therefore, rifampicin and fluconazole were used as perpetrators to induce and inhibit the activities of CYP enzymes, respectively, including CYP3A4, CYP2C8, CYP2C9, and CYP2C19. The AUCR of 2.03 of etrasimod with fluconazole implies a significant increase in the overall drug exposure, suggesting that the inhibition of CYP3A4, CYP2C9, and CYP2C19 together by fluconazole has a significant impact on etrasimod exposure. In contrast, the AUCR of 0.43 with the rifampicin reflects a substantial decrease in drug exposure, which aligns with the expectation that induction of CYP3A4 and CYP2C8 by rifampicin has the potential to accelerate the metabolism of the etrasimod, leading to lower systemic concentrations over time. Regarding C_max_, the ratio of 1.13 with fluconazole suggests an increase in the peak concentration of etrasimod when co-administered with broad CYP enzyme inhibitors such as fluconazole. On the other hand, the C_max_ ratio of 0.70 with rifampicin indicates a reduction in the peak concentration, likely due to the increased clearance of the drug. In addition, the predicted T-half ratio of 1.87 with the enzyme inhibitor indicates a prolonged elimination time due to decreased clearance, while the predicted T-half ratio of 0.40 with the inducer suggests a shortened T-half, reflecting increased metabolism and faster clearance. Overall, ratios of these PK parameters are consistent with expectations, as the fm values for enzymes responsible for etrasimod metabolism indicate a high proportion of the drug’s metabolism, making the drug particularly sensitive to changes in the activities of multiple enzymes simultaneously.

The robustness of the developed PBPK model was good enough to predict the effect of inhibition and induction of CYP enzymes on the total exposure of etrasimod, indicating the applicability of the model to be used for estimating the potential effects of drug interaction with subsequent dosing recommendation.

Because etrasimod is a newly approved drug, few clinical studies were only available for us to train and qualify the model and this is very important to be considered while evaluating the results. However, the current model was stable enough to capture most of the currently available observed data and can be used as a preliminary model pending more clinical PK studies becoming available. Moreover, even though data about exposure to etrasimod in the population with hepatic impairment were not directly taken from the original study, the predicted values were comparable to what has been reported by the etrasimod FDA-approved label. In truth, although this model has been successful in predicting etrasimod kinetic behavior in hepatically impaired patient populations, there is no doubt that it needs to be evaluated and optimized further in the future based on emerging clinical pharmacokinetic studies.

## 5. Conclusions

Our study underscores the critical role of PBPK modeling and simulation in optimizing drug dosing of etrasimod for patients with hepatic impairment. The model effectively predicted the need for dose reductions in patients with Child–Pugh B and C classifications to achieve therapeutic exposure comparable to that in healthy individuals. These findings highlight the utility of PBPK models in personalizing treatment, reducing the risk of adverse effects, and enhancing therapeutic efficacy in this vulnerable population. Future clinical studies are essential to verify these recommendations and further refine dosing strategies for broader clinical use.

## Figures and Tables

**Table 1:** Clinical trials were used for developing and qualifying the PBPK model of etrasimod. Data presented as mean (SD).

Dose	N (male %)	Age (year)	Weight (kg)	AUC__inf_ [ng·h/mL]	C_max_ [ng/mL]	Ref.
**Phase 1, open label, single dose study to build and refine the model**						
1 mg	18 (68.4)	32 (8.1)	75.7 (11.4)	763 (207)	18.8 (3.3)	[26]
**Phase 1, open label, single dose study to build and refine the model ^a^**						
1 mg	19 (73.7)	35.3 (10.4)	73.5 (14.2)	759 (196)	19 (4.4)	[27]
1 mg	19 (36.8)	36.4 (10.6)	77.1 (16.5)	802 (232)	19.5 (5.0)	
2 mg	18 (83.3)	39.4 (9.1)	80.6 (9.5)	1510 (488)	34.2 (11.1)	
**Phase 1, open label, single dose study to build and refine the model**						
2 mg	8 (100)	31 (6.6)	77.3 (11.48)	1900 (596)	42.5 (10.2)	[16]
**Phase 1, randomized, double-blind, SAD study to verify the model**						
0.1 mg	6 (66.7)	27.3 (5.8)	27.8 ± 4.6 (BMI)	79.8 (21.3)	1.7 (0.6)	[28]
0.35 mg	6 (33.3)	25.8 (4.4)	30.0 ± 4.9 (BMI)	268 (31.0)	6.3 (0.4)	
1 mg	6 (50.0)	31.5 (6.4)	29.3 ± 3.8 (BMI)	793 (168)	17.2 (5.5)	
3 mg	6 (66.7)	31.0 (7.1)	27.2 ± 4.7 (BMI)	2600 (840)	60.5 (11.7)	
5 mg	6 (33.3)	33.0 (9.4)	27.2 ± 6.4 (BMI)	4390 (610)	102 (19.1)	
**Phase 1, randomized, double-blind, MAD study to verify the model. PK parameters after the last dose**						
0.7 mg	10 (50.0)	34.2 (8.8)	28.9 ± 4.5 (BMI)	596 (121)	30.8 (6.6)	[28]
1.35 mg	10 (30.0)	31.4 (9.0)	26.9 ± 3.1 (BMI)	1197 (226)	63.5 (11.8)	
2 mg	10 (40.0)	30.1 (7.0)	27.0 ± 5.8 (BMI)	2163 (489)	113 (27.5)	
0.35 mg/2 mg	10 (50.0)	32.8 (6.0)	27.2 ± 2.1 (BMI)	1513 (359)	80.5 (17.4)	
0.5 mg/3 mg	10 (40.0)	29.0 (7.2)	27.7 ± 2.2 (BMI)	2867 (376)	151 (19.0)	
**Phase 1, randomized, double-blind, MAD study to verify the model. PK parameters after the last dose**						
2 mg/3 mg/4 mg	30	18–55	50–100	2885 (793)	163 (46.7)	[29]

^a^ Values represent etrasimod alone from three different cohorts.

**Table 4:** Predicted and observed AUC ratios (AUCR), C_max_ ratios, (C_max_R), and T½ ratios (T½R) for etrasimod as a victim for drug interaction.

Group	Data	AUC__inf_ [ng·h/mL]	C_max_ [ng/mL]	T½ [h]
**Effect of enzymes inhibition**				
Control ^†^	Predicted	875	17.7	44.0
	Observed	759	19.0	42.5
	Fold error	1.15	0.93	1.04
Etrasimod + Fluconazole	Predicted	1772	20.0	82.1
	Observed	1440	21.4	88.2
	Fold error	1.23	0.93	0.70
Pred. AUCR		2.03		
Obs. AUCR		1.90		
Pred/Obs AUCR		1.07		
Pred. C_max_R			1.13	
Obs. C_max_R			1.13	
Pred/Obs C_max_R			1.0	
Pred. T½R				1.87
Obs. T½R				2.08
Pred/Obs T½R				0.90
**Effect of enzymes induction**				
Control ^‡^	Predicted	1750	34.3	44.0
	Observed	1510	34.2	41.1
	Fold error	1.16	1.0	1.10
Etrasimod + Rifampicin	Predicted	754	24.0	17.0
	Observed	770	36.0	21.0
	Fold error	0.98	0.67	0.81
Pred. AUCR		0.43		
Obs. AUCR		0.51		
Pred/Obs AUCR		0.84		
Pred. C_max_R			0.70	
Obs. C_max_R			1.05	
Pred/Obs C_max_R			0.67	
Pred. T½R				0.40
Obs. T½R				0.51
Pred/Obs T½R				0.78

^†^ 1 mg etrasimod alone. ^‡^ 2 mg etrasimod alone.

**Table 5:** Comparison of predicted and observed etrasimod exposure in healthy and hepatically impaired patients.

Parameter	Population	Observed	Predicted	Fold Error
AUC__inf_ (ng·h/mL)	Healthy	1510 ^a^	1750	1.16
	CP-A	1706 (13% ↑) ^b^	1894 (12.50% ↑)	1.11
	CP-B	1948 (29% ↑) ^b^	2208 (28.40% ↑)	1.13
	CP-C	2371 (57%↑) ^b^	2526 (52.4% ↑)	1.07

^a^ [27]. ^b^ FDA approved label of etrasimod (LABEL (fda.gov)) [11].

## Data Availability

The raw data supporting the conclusions of this article will be made available by the authors upon request.
